# PTMs of PD-1/PD-L1 and PROTACs application for improving cancer immunotherapy

**DOI:** 10.3389/fimmu.2024.1392546

**Published:** 2024-04-04

**Authors:** Xiaohui Ren, Lijuan Wang, Likun Liu, Juan Liu

**Affiliations:** ^1^Department of Respiratory Medicine, Shanxi Province Cancer Hospital/Shanxi Hospital Affiliated to Cancer Hospital, Chinese Academy of Medical Sciences/Cancer Hospital Affiliated to Shanxi Medical University, Taiyuan, Shanxi, China; ^2^Department of Hospice Care, Shanxi Province Cancer Hospital/Shanxi Hospital Affiliated to Cancer Hospital, Chinese Academy of Medical Sciences/Cancer Hospital Affiliated to Shanxi Medical University, Taiyuan, Shanxi, China; ^3^Shanxi Province Cancer Hospital/Shanxi Hospital Affiliated to Cancer Hospital, Chinese Academy of Medical Sciences/Cancer Hospital Affiliated to Shanxi Medical University, Taiyuan, Shanxi, China; ^4^Department of Special Needs Medicine, Shanxi Province Cancer Hospital/Shanxi Hospital Affiliated to Cancer Hospital, Chinese Academy of Medical Sciences/Cancer Hospital Affiliated to Shanxi Medical University, Taiyuan, Shanxi, China

**Keywords:** PROTAC, PD-1, PD-L1, immunotherapy, ubiquitination

## Abstract

Immunotherapy has been developed, which harnesses and enhances the innate powers of the immune system to fight disease, particularly cancer. PD-1 (programmed death-1) and PD-L1 (programmed death ligand-1) are key components in the regulation of the immune system, particularly in the context of cancer immunotherapy. PD-1 and PD-L1 are regulated by PTMs, including phosphorylation, ubiquitination, deubiquitination, acetylation, palmitoylation and glycosylation. PROTACs (Proteolysis Targeting Chimeras) are a type of new drug design technology. They are specifically engineered molecules that target specific proteins within a cell for degradation. PROTACs have been designed and demonstrated their inhibitory activity against the PD-1/PD-L1 pathway, and showed their ability to degrade PD-1/PD-L1 proteins. In this review, we describe how PROTACs target PD-1 and PD-L1 proteins to improve the efficacy of immunotherapy. PROTACs could be a novel strategy to combine with radiotherapy, chemotherapy and immunotherapy for cancer patients.

## Introduction

Post-translational modification (PTM) is one kind of the chemical modification of a protein after its synthesis (1). PTM often occurs after the translation process in which ribosomes create proteins from mRNA templates. It has been known that PTMs have profound effects on protein stability, activity, localization, and interaction with other cellular molecules, leading to regulation of the cellular function that was governed by proteins (2). PTMs play a central role in a wide range of biological processes, including cell signaling pathway, immune response, DNA damage response, and metabolism (3, 4). Dysregulation of PTMs is associated with various diseases, including cancer (5–7), neurodegenerative disorders (8, 9), cardiovascular diseases (10), and metabolic syndromes (11, 12). It is critical to explore the regulatory mechanism of PTMs in order to discover the new targets for disease treatments.

The common types of PTMs include ubiquitination (13), acetylation (14, 15), phosphorylation (16), methylation (17), glycosylation (18, 19), SUMOylation (20), prenylation (21), GlcNAcylation (18), succinylation (22), palmitoylation (23), and neddylation (24, 25). Ubiquitination is one common type of PTMs, in which a target protein is added a small protein called ubiquitin or multiple ubiquitins, leading to protein degradation by the proteasome (26). This process involves three key enzymes: E1 (Ubiquitin-activating enzyme), E2 (Ubiquitin-conjugating enzyme) and E3 (Ubiquitin ligase). The E1 enzyme activates ubiquitin in an ATP-dependent manner, forming a high-energy thioester bond with ubiquitin. The activated ubiquitin is transferred to E2, maintaining the high-energy bond. The E3 enzyme facilitates the transfer of ubiquitin from E2 to the substrate protein (27). E3 ligases are responsible for the specificity of the ubiquitination process, as they recognize specific target proteins to be ubiquitinated (28). There are different outcomes of ubiquitination, which are dependent on the number and linkage type of ubiquitin molecules. Monoubiquitination means only single ubiquitin is added to the substrate protein (29). Polyubiquitination means multiple ubiquitin molecules are attached and forma chain (30). The common and well-studied forms are linked through the lysine 48 (K48) residue and lysine 63 (K63) residue of ubiquitin. K48-linked polyubiquitination often tags the substrate protein for degradation by the proteasome, a cellular complex that breaks down proteins (31). K63-linked polyubiquitination often has different roles, such as in signaling pathways, DNA repair, or trafficking of membrane proteins (32). Beyond K48 and K63, non-canonical protein ubiquitination through K6, K11, K27, K29 and K33 residues have been reported (33). K6-linked ubiquitination is involved in autophagy and DNA damage response, while K27-linked ubiquitination participates into innate immunity. K29-linked ubiquitination plays a role in neurodegenerative disorders, whereas K11- and K33-linked ubiquitination should be further investigated (33).

E3 ubiquitin ligases are crucial enzymes in the ubiquitin-proteasome system (UPS). E3 ligases play a pivotal role in the process of ubiquitination, which involves attaching ubiquitin molecules to specific substrate proteins (34). They act as a bridge between the E2 enzyme and the substrate, ensuring accurate ubiquitin transfer. The E3 ligases are primarily responsible for the specificity of ubiquitination, determining which proteins will be tagged for various fates, including degradation, localization, or involvement in various cellular processes (35). There are several types of E3 ligases, categorized mainly based on their mechanism of action and structural features. The common types are RING (Really interesting new gene) finger ligases and HECT (Homologous to E6-AP carboxyl terminus) ligases. RING ligases directly transfer ubiquitin from the E2 enzyme to the substrate. HECT ligases form a covalent bond with ubiquitin before transferring it to the substrate (36).

RING E3 ligases are a diverse group with several types and subtypes, each having unique structural features and mechanisms of action (37). The notable types of RING E3 ligases have single-subunit RING E3 ligases and multiple-subunit E3 ligases. The former type is composed of a single protein that contains both the RING domain necessary for E2 binding and a substrate-recognition domain, such as MDM2. MDM2 targets the tumor suppressors p53, and c-Cbl. The latter type are complexes made up of multiple protein subunits. One of the subunits contains the RING domain, while others are responsible for substrate recognition and regulation. Multiple-subunit E3 ligases mainly have cullin-RING ligases (CRLs) and RBR (RING-between-RING) E3 Ligases (38). CRLs are the largest family of multi-subunit RING E3 ligases, which include several subfamilies like SCF (Skp1-Cullin1-F-box protein) complexes. CRLs are involved in various cellular processes including cell cycle control and signal transduction (39). RBR are a unique class of E3 ligases that contain two RING domains separated by a non-RING domain. They function through a hybrid mechanism that has characteristics of both RING and HECT type E3 ligases (40).

Unlike RING E3 ligases, HECT E3 ligases form a covalent bond with ubiquitin before transferring it to the substrate protein. This family is characterized by the HECT domain, which is responsible for this unique enzymatic activity (41). HECT ligases are classified into different types based on structural features and functional domains. HECT ligases have several notable types, including NEDD4 family and HERC family (42). Members of this family typically have a C2 domain that binds to phospholipids, two to four WW domains that recognize proline-rich motifs in substrates, and the HECT domain for ubiquitin transfer (43). NEDD4 family is the largest group of HECT E3 ligases and is composed of nine members, each with distinct but sometimes overlapping substrate specificities and functions (44). These members typically include NEDD4 (Neural Precursor Cell Expressed Developmentally Downregulated 4), NEDD4L (NEDD4-Like), ITCH (Itchy E3 Ubiquitin Protein Ligase), WWP1 (WW Domain Containing E3 Ubiquitin Protein Ligase 1), WWP2, SMURF1 (SMAD Specific E3 Ubiquitin Protein Ligase 1), SMURF2, NEDL1 (NEDD4-Like E3 Ubiquitin Protein Ligase 1) and NEDL2 (45–48). HERC E3 ligases are characterized by their large size and the presence of one or more RCC1-like domains (RLDs) in addition to the HECT domain. The HERC family is involved in processes such as protein trafficking and cellular growth control (49, 50).

Ubiquitination is a dynamic and reversible process, with deubiquitinating enzymes (DUBs) able to reverse the ubiquitination of proteins (51). This process, known as deubiquitination, is essential for maintaining the balance and regulation of protein ubiquitination within the cell, impacting various cellular processes and homeostasis (52, 53). DUBs are a group of proteases that play a critical role in the UPS by removing ubiquitin molecules from substrate proteins. Different DUBs have specificity for different types of ubiquitin linkages. Some are specialized in trimming ubiquitin chains from the distal end, while others can cleave ubiquitin at specific linkage points within a chain. There are several classes of DUBs, based on their structural and functional characteristics (54). The two main classes are cysteine proteases and metalloproteases. These classes are further divided into families such as USP (ubiquitin-specific protease), UCH (ubiquitin C-terminal hydrolase), OTU (ovarian tumor protease), and JAMM/MPN+ (Jab1/MPN domain-containing metalloenzymes) (55–58).

## PROTACs

**Figure 1:**
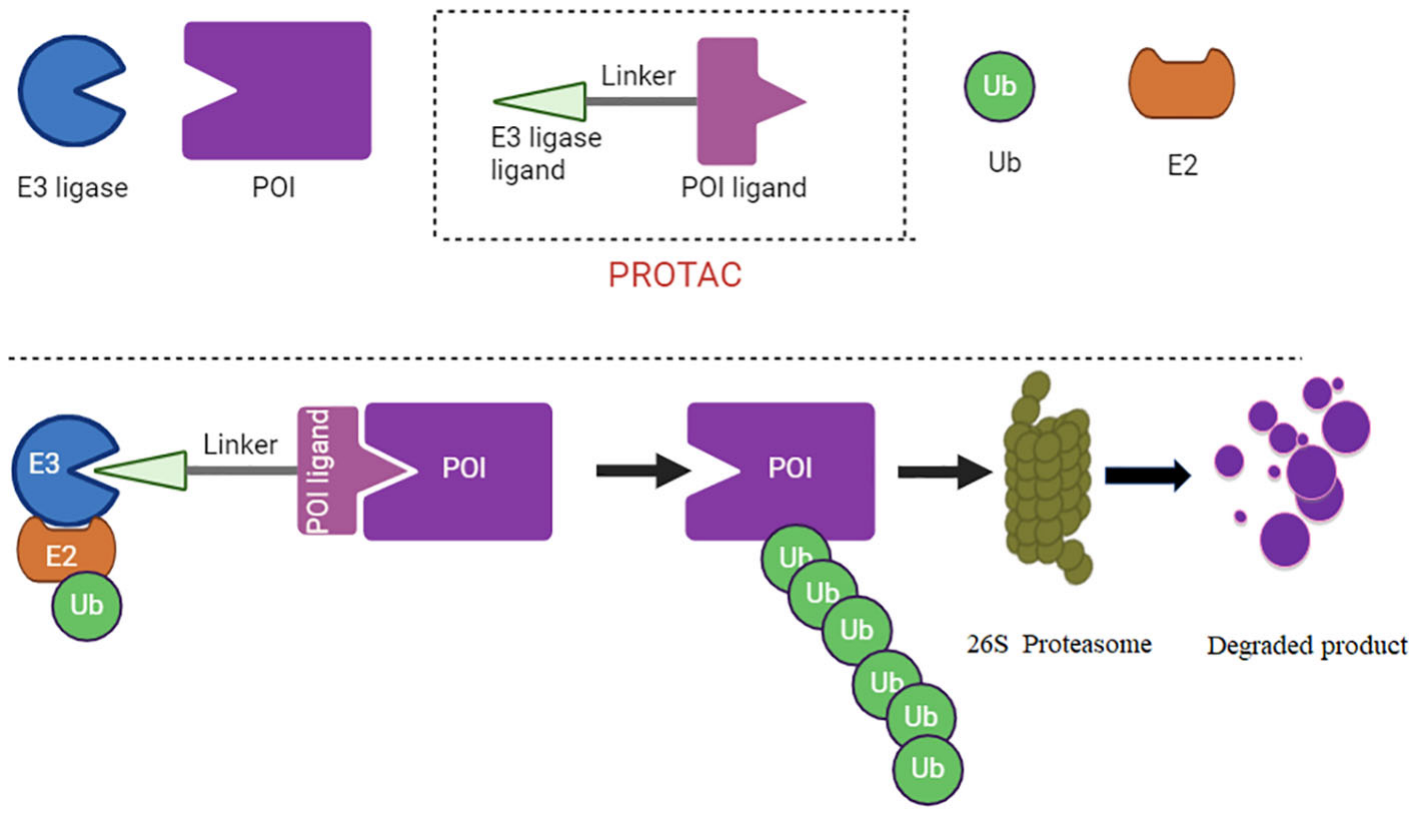
PROTAC diagram is illustrate. The PROTAC recruits an E3 ubiquitin ligase, which is responsible for transferring ubiquitin molecules to the target protein, tagging it for degradation. The linker connects the target protein ligand and the E3 ligase ligand.

PROTACs (Proteolysis Targeting Chimeras) are a type of new drug design technology. They are specifically engineered molecules that target specific proteins within a cell for degradation (59). PROTACs consist of three key components: a target protein ligand, a E3 ubiquitin ligand, and a linker. Target protein ligand is the part of the PROTAC molecule that specifically binds to the protein targeted for degradation (60). The selection of this ligand is critical as it ensures the specificity of the PROTAC for its intended protein target (61). The PROTAC recruits an E3 ubiquitin ligase, which is responsible for transferring ubiquitin molecules to the target protein, tagging it for degradation. The linker connects the target protein ligand and the E3 ligase ligand (**Figure 1**). The length and composition of the linker are crucial for the effective proximity of the target protein and the E3 ligase, facilitating the transfer of ubiquitin from the E3 ligase to the target protein (62, 63).

Unlike traditional inhibitors that simply block protein activity, PROTACs work by recruiting an E3 ubiquitin ligase to tag the target protein with ubiquitin. This tag marks the protein for destruction by the cell’s proteasome, a protein complex responsible for degrading and recycling damaged or unneeded proteins (64). PROTAC technology allows for more precise control targeting protein levels within cells, which can be used to target proteins that are traditionally considered “undruggable” by conventional methods (65). AbTAC, molecular glue, LYTAC and Nano-PROTAC have been emerged to target proteins (66). PROTACs have been gaining attention in the field of drug development, particularly for their potential in treating diseases such as cancer, where certain proteins are overexpressed or mutated (67, 68).

## Immunotherapy

Immunotherapy has been developed, which harnesses and enhances the innate powers of the immune system to fight disease, particularly cancer (69). Unlike chemotherapy and radiation, which directly target and kill cancer cells, immunotherapy performs its function via stimulating or restoring the immune system’s ability to detect and destroy tumor cells (70, 71). There are several types of immunotherapy to boost the immune response via employing a different strategy (72). Checkpoint inhibitors were discovered, which block proteins that prevent immune cells from attacking cancer cells (73). By inhibiting these “checkpoints”, checkpoint inhibitors enable the immune cells to recognize and destroy cancer cells, including inhibitors targeting PD-1, PD-L1, and CTLA-4 (74).

CAR T-cell therapy (Chimeric Antigen Receptor T-cell Therapy) involves genetically engineering a patient’s own T cells to produce special receptors on their surface (75, 76). These receptors will make T cells to better recognize and attack cancer cells. Cancer vaccines are designed to treat existing cancers by stimulating the immune system to attack cancer cells, such as targeting specific antigens found on cancer cells (77). Cytokine therapy boosts the immune system’s ability to fight cancer via using cytokines, such as interferons and interleukins (78, 79). Immunotherapy has shown remarkable overcomes in treating certain types of cancer, including melanoma, lung cancer, and leukemia. However, its effectiveness can vary widely among various cancer patients. In addition, immunotherapy could have side effects due to an overactive immune response and resistance (80). Therefore, immunotherapy represents a significant shift in cancer treatment, moving toward more personalized and targeted approaches that leverage the body’s natural defenses.

## PD-1 and PD-L1

PD-1 (programmed death-1) and PD-L1 (programmed death ligand-1) are key components in the regulation of the immune system, particularly in the context of cancer immunotherapy (81). Their interaction plays a significant role in preventing the immune system from attacking normal cells, but it can also enable cancer cells to evade immune surveillance (82, 83). PD-1 is a protein receptor expressed on the surface of certain immune cells, such as T cells, B cells, and natural killer cells (84). PD-L1 is a ligand that is expressed on the surface of many cell types, including some cancer cells and immune cells (85). Under normal conditions, PD-L1 binding to PD-1 sends an inhibitory signal to T cells, telling them not to attack the cells presenting PD-L1. This is a natural mechanism to prevent autoimmunity and control inflammation (86). Many types of cancer cells exploit this pathway by overexpressing PD-L1. When cancer cells present high levels of PD-L1, they can bind to PD-1 receptors on T cells, effectively “turning off” these immune cells and preventing them from attacking the cancer (87). Given the role of the PD-1/PD-L1 pathway in allowing cancer cells to evade the immune system, blocking this interaction has become a key strategy in cancer immunotherapy (88). Drugs known as checkpoint inhibitors have been developed to target PD-1, PD-L1 and CTLA-4 (89, 90).

PD-1 inhibitors bind to PD-1 on immune cells, and block its interaction with PD-L1, such as nivolumab and pembrolizumab (91). PD-1 inhibitors allow the T cells to remain active and able to attack cancer cells. PD-L1 Inhibitors bind to PD-L1 on cancer cells or other cells in the tumor environment, which prevent it from binding to and inhibiting PD-1 on T cells. PD-L1 inhibitors include atezolizumab and durvalumab (92). PD-1 and PD-L1 inhibitors have shown significant success in treating various types of cancer, including melanoma, non-small cell lung cancer, kidney cancer, bladder cancer, and head and neck cancers. Although the development and use of PD-1 and PD-L1 inhibitors represent a major advancement in cancer therapy, offering hope for many patients with advanced or hard-to-treat cancers, side effects and resistance reduced the efficacy of PD-1/PD-L1 inhibitors in immunotherapy (93, 94).

## PD-1 and PD-L1 are regulated by PTMs

It has been reported that PD-1 and PD-L1 are regulated by PTMs, including phosphorylation, ubiquitination, deubiquitination, acetylation, palmitoylation and glycosylation (95–97). PD-1/PD-L1 modification participated in governing immune escape and affecting cancer immunotherapy (98). Moreover, the E3 ubiquitin ligases improved tumor immunotherapy via controlling PD-1/PD-L1 protein accumulation in tumor microenvironment (99). In the following paragraphs, we will describe the role of PTMs in regulation of PD-1 and PD-L1 (**Tables 1**, **2**).

**Table 1:** Ubiquitination and deubiquitination of PD-1/PD-L1 are summarized.

PTM	Targets	Functions	Reference
SPOP	Cyclin D/CDK4 causes PD-L1 destabilization through SPOP-mediated PD-L1 degradation.	Cyclin D/CDK4 controls cancer immune surveillance.	(100)
SPOP	RIG-I modulates SPOP-mediated degradation of PD-L1.	RIG-I elevates immune evasion in colon cancer.	(101)
FBXO38	FBXO38 induces ubiquitination of PD-1.	FBXO38 controls anti-tumor immunity of T cells	(102)
NEDD4	NEDD4 mediates PD-L1 ubiquitination and degradation.	NEDD4 regulates T cell-induced immune surveillance in bladder cancer.	(103)
RNF125	RNF125 promote the degradation of PD-L1 in K48-linked manner.	RNF125 was overexpressed, PD-L1 levels decreased, and tumor growth slowed.	(104)
RNF125	RNF125 targets PD-L1 expression.	RNF125 suppresses immune escape in head and neck squamous cell carcinoma.	(105)
TRIM21	TRIM21 mediates PD-L1 ubiquitination.	TRIM21 involves in regulation of anti-PD-L1 immunotherapeutic efficacy.	(106)
TRIM21	CDK5 inhibition reduced PD-L1 levels by TRIM21.	CDK5 inhibition improves antitumor immunity in lung adenocarcinoma.	(107)
TRIM21	TRIM21 restricts the expression of PD-1 in lymphocytes and PD-L1 in tumors.	TRIM21 limits the emergence of HCC nodules in mice with NASH.	(108)
c-Cbl	c-Cbl induces proteasomal degradation of PD-1 in immune cells.	c-Cbl regulates colorectal tumor growth.	(109)
FBW7	FBW7 promotes PD-1 destruction in non-small cell lung cancer.	FBW7 enhanced sensitivity of anti-PD-1 immunotherapy.	(110)
USP22	USP22 deubiquitinated PD-L1 and maintained its stabilization.	USP22 inhibits anticancer immunity.	(111, 112)
USP7	USP7 regulates PD-L1 stability.	USP7 modulates sensitization of gastric cancer cells to T cells killing.	(113)
USP7	USP7 inhibitors upregulated the expression of PD-L1 in tumors.	USP7 reprogrammed macrophages and modulates antitumor immune response in lung cancer.	(114)
USP7	USP7 contributed to the stabilization of PD-L1.	Knocking down USP7 in glioma cells enhanced CD8+ T cell proliferation, prevented immune evasion.	(115)
USP8	USP8 inhibition elevates PD-L1 abundance via elevating TRAF6-induced ubiquitination of PD-L1.	USP8 inhibition reshapes an inflamed TME that enhances the immunotherapy.	(116)
USP8	USP8 deubiquitinates PD-L1.	Targeting USP8 sensitized anti-PD-L1 immunotherapy.	(117)
OTUB1	circIGF2BP3 stabilizes OTUB1 mRNA and inhibits PD-L1 degradation.	circIGF2BP3 regulates tumor immune escape.	(118)
OTUB2	OTUB2 regulates PD-L1 degradation.	Targeting OTUB2 increased cytotoxic T cells efficacy.	(119)

**Table 2:** PTMs of PD-1/PD-L1 are summarized.

PTM	Targets	Functions	Reference
Phosphorylation	IL-6/JAK1 directed PD-L1 phosphorylation at Y112 site.	Promotes tumor immune evasion.	(120)
Phosphorylation	CK2 mediated PD-L1 phosphorylation at the Thr285 and Thr290 and stability.	Inhibits biological function of dendritic cells.	(121)
Phosphorylation	circ_0136666 regulates PD-L1 phosphorylation and miR-375/PRKDC pathway.	Promotes tumor development and immune escape in gastric cancer.	(122)
Acetylation	p300 mediates PD-L1 acetylation, while HDAC2 mediates deacetylation of PD-L1.	Targeting PD-L1 acetylation increases efficacy of immunotherapy.	(123)
Acetylation	HBXIP interacts with p300 and promotes PD-L1 acetylation and stability.	PD-L1-induced tumor growth was retarded by HBXIP downregulation.	(124)
Palmitoylation	Stabilizes PD-L1.	Contributes to promotion of tumor growth in breast cancer.	(125)
Palmitoylation	Stabilizes PD-L1 by blocking its ubiquitination and degradation by lysosomes.	Suppression of PD-L1 palmitoylation increases T cell immune efficacy against tumors.	(126)
Palmitoylation	One peptidic inhibitor targets PD-1 palmitoylation and	One peptidic inhibitor blocks PD-1 expression and its biological functions	(127)
Palmitoylation	ZDHHC9 downregulation enhances the degradation of PD-L1 via reducing its palmitoylation.	Regulates anti-tumor immunity and growth of lung cancer cells.	(128)
Glycosylation	ISG15 influenced glycosylated PD-L1 and induced its destruction.	ISG15 increases antitumor immune functions in lung adenocarcinoma.	(129)
Glycosylation	MDM2 governs degradation of PD-1 through regulating crosstalk between ubiquitination and deglycosylation.	Stimulating the p53-MDM2, IFN-α reduces PD-1 levels in T cells, exhibits sensitizing anti-PD-1 immunotherapy.	(130)
Glycosylation	GLT1D1 regulates PD-L1 glycosylation.	GLT1D1 upregulation causes immunosuppression.	(131)
Glycosylation	PD-1 glycosylation promoted the binding of cemiplimab.	Affects the efficacy of immune checkpoint inhibitors.	(132)
Glycosylation	TGF-β1 induced PD-L1 glycosylation.	TGF-β1 causes immune escape via regulating Jun/STT3A in nasopharyngeal carcinoma.	(133)
UFMylation	UFL1 ablation in T cells suppresses PD-1 UFMylation.	UFL1 enhances anti-tumor immunity.	(134)
UFMylation	UFMylation of PD-L1 destabilizes PD-L1 by synergizing its ubiquitination.	PD-L1 dysregulation by UFMylation regulates tumor immune evasion.	(135)

### Phosphorylation

PD-L1 phosphorylation has been found to regulate cancer immune evasion. For example, IL-6/JAK1 signaling pathway directed PD-L1 phosphorylation at Tyr112 site and promoted tumor immune evasion (120). When IL-6 activated JAK1, it led to the phosphorylation of PD-L1 at Tyr112. This phosphorylation attracted the N-glycosyltransferase STT3A from the endoplasmic reticulum, which initiated the glycosylation of PD-L1, thereby stabilizing it. By using an IL-6 antibody to inhibit IL-6, enhanced T cell killing effects in animal models were reported, especially when used in combination with anti-Tim-3 therapy. Furthermore, a direct relationship between the levels of IL-6 and PD-L1 was found in the tumor tissues of patients with hepatocellular carcinoma (120).

Another study revealed that in both cancer and dendritic cells (DC), PD-L1 underwent phosphorylation and subsequent stabilization by CK2 enzyme. This phosphorylation at the Thr285 and Thr290 sites on PD-L1 interfered with its binding to the adaptor protein, SPOP (speckle-type POZ protein). As a result, PD-L1 was shielded from CUL3/SPOP-mediated proteasomal degradation. When CK2 activity was hindered, there was a noticeable reduction in PD-L1 protein levels, facilitating the mobilization of CD80 from DC, which subsequently revitalized T-cell activities. Using a treatment combination of a CK2 inhibitor and a Tim-3 antibody in a mouse model, marked suppression of tumor growth and a signification prolongation of survival were reported. Hence, CK2-mediated PD-L1 phosphorylation and stability inhibited biological function of dendritic cells (121).

Recently, Miao et al. reported that circ_0136666 regulated PD-L1 phosphorylation and miR-375/PRKDC pathway, leading to promoting tumor development and immune escape in gastric cancer (122). Specifically, the expression of hsa_circ_0136666 was prominent in gastric cancer tissues and cell lines. Functionally, hsa_circ_0136666 enhanced the proliferation of gastric cancer and contributed to the development of the tumor microenvironment, enabling the tumor to evade immune surveillance through the modulatory activities on CD8+ T cells. At the mechanistic level, hsa_circ_0136666 induced the increase of PRKDC expression by competitively binding to miR-375-3p, affecting the phosphorylation and subsequent stabilization of PD-L1. Moreover, hsa_circ_0136666 suppressed the body’s immune vigilance and countering anti-cancer efficacy. Furthermore, lipid nanoparticle (LNP)-delivered siRNA strategies markedly improved anti-PD-L1 efficacy and inhibited immune escape (122).

### Ubiquitination

#### SPOP targets *PD-L1*

Zhang et al. found that Cyclin D/CDK4 kinase leads to PD-L1 destabilization through SPOP-mediated degradation of PD-L1 and controls cancer immune surveillance (100). The levels of the PD-L1 protein are controlled through the actions of cyclin D-CDK4 and the CUL3-SPOP E3 ligase, which facilitate its degradation via the proteasome. Blocking the activity of CDK4 and CDK6 *in vivo* leads to an elevation in PD-L1 protein. This increase is due to the inhibition of the phosphorylation by cyclin D-CDK4 on the SPOP, which in turn enhances the breakdown of SPOP by the FZR1. Furthermore, mutations that result in a loss of SPOP function disrupt the PD-L1 degradation, resulting in heightened levels of PD-L1 and a decrease in the number of tumor-infiltrating lymphocytes in both mouse tumors and prostate cancer tissues. Importantly, CDK4/6 inhibitor therapy plus anti-PD-1 immunotherapy significantly boosts tumor shrinkage and substantially enhances survival rates in mouse models of cancer (100).

RIG-I modulated SPOP-mediated degradation of PD-L1 and elevated immune evasion in colon cancer (101). Suppressing RIG-I led to a decrease in the ability of T cells to eliminate tumor cells and reduced the growth of colon tumors in mice with a fully functional immune system. Conversely, increasing RIG-I expression accelerated tumor growth, and high levels of RIG-I made cells more responsive to anti-PD-1 treatment *in vivo*. Intriguingly, RIG-I modulates the expression of PD-L1, facilitating immune escape in colon cancer independently of type I interferon activation. RIG-I prevents PD-L1 destruction by SPOP (101).

#### FBXO38 targets PD-1

Meng et al. found that FBXO38 induced ubiquitination of PD-1 and controlled anti-tumor immunity of T cells (102). PD-1 on the surface of activated T cells was internalized, then underwent ubiquitination and proteasome-mediated degradation. FBXO38 targeted PD-1 for Lys48-linked polyubiquitination, leading to its degradation. Eliminating Fbxo38 in T cells accelerated tumor growth in mice due to increased PD-1 levels in the tumor-infiltrating T cells. Applying anti-PD-1 therapy counteracted the tumor growth observed with FBXO38 deficiency, indicating that PD-1 is a crucial target of FBXO38 in T cells. In both human tumor samples and a mouse cancer model, the expression levels of FBXO38 and Fbxo38 were reduced in tumor-infiltrating T cells. Nonetheless, IL-2 treatment was able to boost Fbxo38 expression, thus lowering PD-1 levels in PD-1-positive T cells in mice. FBXO38 controlled PD-1 levels, suggesting a novel strategy for inhibiting the PD-1 pathway by regulation of FBXO38 (102).

#### NEDD4 targets PD-L1

NEDD4 mediated PD-L1 ubiquitination and degradation and regulated T cell-induced immune surveillance in bladder cancer, which was regulated by fibroblast growth factor receptor 3 (FGFR3) (103). Blocking FGFR3 in bladder cancer with FGFR3 activation increased PD-L1 protein levels by altering its ubiquitination process, which in turn, hampered the anticancer activity of CD8+ T cells. An analysis of tissue microarrays from human urothelial carcinoma (UC) revealed a negative relationship between FGFR3 expression and PD-L1 levels. Additionally, NEDD4 became phosphorylated upon FGFR3 activation, playing a pivotal role in controlling PD-L1 ubiquitination. NEDD4 directly interacted with PD-L1, leading to the polyubiquitination of PD-L1 at the Lys48 (K48) linkage. In mice models with NEDD4-deficient bladder cancer, an increase in PD-L1 levels within the cancer cells resulted in reduced infiltration and antitumor activity of CD8+ T cells. In various FGFR3-activated tumor models, the diminished antitumor effectiveness due to targeted FGFR3 therapy could be counterbalanced by combining it with anti-PD-1 immunotherapy, thus significantly reducing tumor growth. NEDD4 could be a key E3 ubiquitin ligase that regulates PD-L1 for destruction in FGFR3-driven bladder cancer (103).

#### RNF125 targets PD-L1

Ubiquitin ligase RNF125 was reported by Wei et al. to promote the ubiquitination and degradation of PD-L1 in K48-linked manner (104). RNF125 binds to PD-L1, influencing its protein levels by facilitating K48-linked polyubiquitination, leading to its degradation. When RNF125 was knocked out in MC-38 and H22 cell lines, which were then implanted into C57BL/6 mice, an increase in PD-L1 levels and accelerated tumor growth were observed. Conversely, when RNF125 was overexpressed in these cell lines, PD-L1 levels decreased, and tumor growth slowed. Furthermore, MC-38 tumors with RNF125 overexpression had an increased in the infiltration of CD4+, CD8+ T cells, and macrophages. A positive association between RNF125 expression and the infiltration of CD4+, CD8+ T cells, and macrophages was observed in tumor tissues from TCGA public database. Additionally, RNF125 expression was found to be notably reduced in various human cancer tissues, inversely related to the clinical stage of the cancers, and tumor patients with elevated RNF125 levels displayed more favorable clinical outcomes (104).

In head and neck squamous cell carcinoma (HNSCC) cells, RNF125 expression was low. When RNF125 was overexpressed, it curtailed the immune evasion of HNSCC cells, as demonstrated by reduced proliferation, migration, and invasion of TSCCA cells, alongside enhanced proliferation of CD8+ T cells and increased levels of IL-2 and TNF-α. RNF125 also reduced PD-L1 expression in TSCCA cells and promoted its degradation. Overexpression of PD-L1 partially reversed the effects of RNF125 on the immune evasion of TSCCA cells. Additionally, RNF125 suppressed tumor formation and growth in mice. Collectively, RNF125 facilitates the ubiquitin-mediated degradation of PD-L1, thereby impeding immune escape in HNSCC (105).

#### TRIM21 targets PD-L1

Sun and coworkers reported that TRIM21 mediated PD-L1 ubiquitination and involved in regulation of anti-PD-L1 immunotherapeutic efficacy. LINC02418 acted as a suppressor of PD-L1 expression and was associated with increased CD8+ T cell infiltration, indicating better clinical outcomes for NSCLC patients. It reduced PD-L1 levels by promoting its ubiquitination through TRIM21. Both human LINC02418 and its mouse equivalent, mmu-4930573I07Rik, influenced the effectiveness of PD-L1-targeted therapies in NSCLC by facilitating T cell-mediated apoptosis. Additionally, the suppression of METTL3 by YTHDF2 led to the upregulation of hsa-LINC02418 and mmu-4930573I07Rik. In NSCLC patients, high levels of LINC02418 are linked to lower PD-L1 expression and a higher presence of CD8+ T cells (106). Inhibition of CDK5 reduced PD-L1 levels via the ubiquitination-proteasome pathway by TRIM21 and improved antitumor immunity in lung adenocarcinoma (107). Silencing Trim21 in mice increased HCC oncogenesis in a non-alcoholic steatohepatitis (NASH) context, which was due to overexpression of PD-1 in lymphocytes and PD-L1 in tumors (108).

#### c-Cbl targets PD-1

One group showed that casitas B lymphoma (c-Cbl) induced proteasomal degradation of PD-1 in immune cells and regulated colorectal cancer (CRC) growth (109). A significantly increased growth of xenografts and enhanced infiltration of immune cells in c-Cbl heterozygous (c-Cbl^+/-^) mice was observed compared to c-Cbl^+/+^. Tumor-associated CD8+ T-lymphocytes and macrophages in c-Cbl^+/-^ mice exhibited upregulation of PD-1 levels. Macrophages from c-Cbl^+/-^ mice displayed a 4-5 times decrease in their ability to phagocytize tumor cells, which was recovered by the application of an anti-PD-1 neutralizing antibody, indicating a regulatory role of c-Cbl on PD-1. The C-terminus of c-Cbl interacted with the cytoplasmic tail of PD-1, leading to PD-1 destabilization. Hence, c-Cbl could be as an E3 ligase for PD-1 and a modulator of the tumor microenvironment (109).

#### FBW7 targets PD-L1

Liu et al. reported that FBW7 enhanced sensitivity of anti-PD-1 immunotherapy via promotion of PD-1 ubiquitination and destruction in non-small cell lung cancer (110). FBW7 was identified to act as an E3 ubiquitin ligase targeting the PD-1 protein, specifically promoting K48-linked polyubiquitination at the Lys233 residue of PD-1. Targeting FBW7 caused faster degradation of the PD-1 protein, thereby boosting antitumor immunity effectively *in vivo*. Additionally, phosphorylation of the Ser261 residue by CDK1 enhanced the PD-1 protein for nuclear translocation and interaction with FBW7. A higher level of FBW7 was found in immunologically active TME, resulting in improved responses to PD-1 blockade therapy (110). Hence, ubiquitination plays a critical role in the regulation of PD-1/PD-L1 in tumor immunotherapy (136).

### Deubiquitination

#### USP22 targets PD-L1

Huang et al. reported that USP22 deubiquitinated PD-L1 and maintained its stabilization, leading to inhibition of anticancer immunity. USP22 was identified as a deubiquitinase for PD-L1, where it binds directly to PD-L1’s C-terminus, leading to its deubiquitination and stabilization. USP22 showed high expression levels and frequent alterations in liver cancer, which was strongly linked to a dismal prognosis for these cancer patients. The genetic removal of USP22 led to the suppression of liver cancer growth, boosted tumor immunogenicity and the presence of tumor-infiltrating lymphocytes, and heightened the effectiveness of therapies targeting PD-L1 and CDDP-based chemotherapy in mouse models (111). Another study also showed that USP22 interacted with PD-L1, enhancing its stability by removing ubiquitin and preventing its breakdown by the proteasome. Additionally, USP22 forms a complex with CSN5, maintaining its stability via deubiquitination. USP22 or CSN5 can enhance the binding between PD-L1 and the other molecule. The reduction of USP22 levels was found to suppress tumor growth and enhance the cytotoxicity of T cells. Moreover, in samples from patients with non-small cell lung cancer, there was a notable positive relationship between the levels of USP22 and PD-L1 expression (112). Hence, USP22 plays a critical role in immune evasion in human cancer cells (137).

#### USP7 targets PD-L1

USP7 was reported to regulate PD-L1 stability and modulate sensitization of gastric cancer cells to T cells killing. Analyzing data from TCGA and tissues, a direct correlation between PD-L1 and USP7 expressions was found in gastric cancer. USP7 interacted with PD-L1, enhancing its stability, whereas inhibiting USP7 reduced the PD-L1/PD-1 interaction and made cancer cells more susceptible to T cell-mediated destruction. Additionally, USP7 inhibitors reduced cell growth by stabilizing p53 in gastric cancer. USP7 inhibitors not only hinder the cell proliferation but also reduce PD-L1 levels, thereby boosting the anti-tumor immune response (113). USP7 reprogrammed tumor-associated macrophages and modulates anti-tumor immune response in lung cancer. USP7 inhibitors upregulated the expression of PD-L1 in tumors, while inhibiting PD-1 had an effective anti-tumor activity (114). In glioma cells, USP7 showed high expression levels, but PD-L1 protein levels increased. Knocking down USP7 in glioma cells reduced their growth, increased apoptosis, and enhanced CD8+ T cell proliferation, thereby preventing immune evasion. USP7 contributed to the stabilization of PD-L1. The overexpression of PD-L1 counteracted the effects of USP7 silencing on the immune escape of glioma cells (115).

#### USP8 targets PD-L1

Blocking the USP8 significantly boosts the effectiveness of anti-PD-1/PD-L1 immunotherapy by altering the TME. Inhibiting USP8 elevated PD-L1 protein levels by promoting TRAF6-mediated K63-linked ubiquitination of PD-L1. USP8 blockade enhances the innate immune response and MHC-I expression via the activation of NF-κB signaling. Combining a USP8 inhibitor with PD-1/PD-L1 blockade markedly stimulates CD8+ T cell infiltration, leading to tumor suppression and improved survival in various mouse tumor models (116). In pancreatic cancer, USP8 levels were significantly higher in tumor tissues compared to normal tissues. The expression of USP8 was notably linked to the TNM stage in various pancreatic cancer patient cohorts. Inhibition of USP8 led to diminished tumor invasion, migration, and overall tumor size, enhancing anti-tumor immunity. USP8 inhibitor resulted in decreased tumor formation, and mice with Usp8 deficiency showed improved survival rates. Additionally, USP8 positively influenced PD-L1 expression by blocking its degradation in pancreatic cancer. A USP8 inhibitor and anti-PD-L1 together significantly halted pancreatic tumor growth through the activation of cytotoxic T-cells. The effectiveness of this anti-tumor immunity was primarily reliant on the PD-L1 pathway and CD8+ T cells. Hence, targeting USP8 sensitized anti-PD-L1 immunotherapy via regulation of PD-L1 degradation in pancreatic cancer (117).

#### OTUB1 targets PD-L1

CircRNA circIGF2BP3 stabilized OTUB1 mRNA and inhibited the ubiquitination and degradation of PD-L1 in a PKP3-dependent way, leading to tumor immune escape (118). circIGF2BP3 increases PKP3 expression by sequestering miR-328-3p and miR-3173-5p, thus impairing the cancer immune response. PKP3 then interacts with the RNA-binding protein FXR1 to enhance the stability of OTUB1 mRNA, which in turn boosts PD-L1 levels by promoting its deubiquitination. The elimination of tumor PD-L1 effectively nullified the influence of the circIGF2BP3/PKP3 axis on the CD8+ T cell response. Blocking the circIGF2BP3/PKP3 pathway improved the efficacy of anti-PD-1 treatment in a Lewis lung carcinoma mouse model (118).

#### OTUB2 targets PD-L1

Recently, targeting OTUB2 increased cytotoxic T cells efficacy via regulating PD-L1 degradation. OTUB2 directly bound to PD-L1, hindering its ubiquitination and subsequent degradation within the endoplasmic reticulum. Eliminating OTUB2 significantly reduced PD-L1 protein levels on tumor cells, enhancing their vulnerability to the cytotoxic effects of CD8+ T cells. A notable association between OTUB2 levels and PD-L1 was found in human non-small cell lung cancer samples. An OTUB2 inhibitor effectively diminished PD-L1 levels and inhibited tumor growth. OTUB2 plays a key role in regulating PD-L1 expression and tumor immune evasion (119).

### Acetylation

PD-L1 acetylation has been uncovered by Gao and coworkers in 2020. This study showed that p300 mediated PD-L1 acetylation, while HDAC2 mediated deacetylation of PD-L1. Genetically or pharmacologically modulating acetylation of PD-L1 reduced its nuclear translocation, which promoted the anti-PD-1 efficacy via regulation of the expression of several immune-response-related genes in cancer cells. Targeting PD-L1 acetylation could increase the efficacy of tumor immunotherapy (123). Another study showed that HBXIP oncoprotein and PD-L1 expressions were upregulated in clinical breast tumor samples. Moreover, HBXIP expression was associated with PD-L1 expression in breast cancer tissues. Mechanistically, HBXIP elevated the PD-L1 transcription via activation of ETS2. Furthermore, HBXIP interacted with p300 and promoted PD-L1 acetylation and resulted in promotion of PD-L1 stability. PD-L1-induced tumor growth was retarded by downregulation of HBXIP in breast cancer (124).

### Palmitoylation

Palmitoylation has been discovered to stabilize PD-L1 and contribute to promotion of tumor growth in breast cancer (125). Yao et al. reported that suppression of PD-L1 palmitoylation increased T cell immune efficacy against tumors (126). One peptidic inhibitor has been developed for targeting PD-1 palmitoylation and blocking PD-1 expression and its biological functions (127). Evidence showed that downregulation of ZDHHC9 enhanced the degradation of PD-L1 protein via reducing its palmitoylation levels in lung adenocarcinoma (128). Hence, PD-1/PD-L1 palmitoylation is involved in immunotherapy in human cancers.

### Glycosylation

N-glycosylation and stabilization of PD-L1 were reported to inhibit T-cell activity. Glycogen synthase kinase 3β (GSK3β) can bind to PD-L1 and promote PD-L1 degradation by beta-TrCP in phosphorylation-dependent manner. Moreover, epidermal growth factor (EGF) treatment made PD-L1 stabilization due to inactivation of GSK3β in breast cancer. Consistently, gefitinib destabilized PD-L1 due to suppression of EGF pathway, which increased T-cell immunity and promoted PD-1 blockade efficacy in mice (138). ISG15 influenced glycosylated PD-L1 and induced its destruction to increase antitumor immune functions in lung adenocarcinoma (129). MDM2 (murine double minute 2) governed degradation of PD-1 through regulating crosstalk between ubiquitination and deglycosylation in tumor cells (130). Removal of N-glycosylation increased the detection of PD-L1 and provided the prediction of anti-PD-1/PD-L1 treatment efficacy (139). GLT1D1 upregulation caused immunosuppression via regulation of PD-L1 glycosylation and directed poor prognosis in B-cell lymphoma (131). N-glycosylation of PD-1 enhanced the interaction with a PD-1-specific monoclonal antibody, camrelizumab (140). PD-1 glycosylation promoted the binding of cemiplimab to affect the efficacy of immune checkpoint inhibitors (132). TGF-beta-1 induced PD-L1 glycosylation and led to immune escape via regulation of Jun/STT3A pathway in nasopharyngeal carcinoma (133).

### UFMylation

UFMylation, a post-translational modification akin to ubiquitination, plays a crucial role in various biological functions, with its dysregulation linked to several human diseases, including cancer (141, 142). Mice lacking the UFMylation E3 ligase UFL1 specifically in T cells demonstrated superior tumor suppression. Through single-cell RNA sequencing, an increase in tumor-infiltrating cytotoxic CD8+ T cells was observed in these UFL1 conditional knockout (cKO) mice. UFL1 was found to enhance PD-1 stability by promoting its UFMylation, which counters PD-1 ubiquitination and subsequent degradation (134). Moreover, AMPK activation led to the phosphorylation of UFL1 at Thr536, which inhibited PD-1 UFMylation and promoted its degradation. Notably, the removal of UFL1 in T cells led to reduced PD-1 UFMylation, destabilizing PD-1 and thereby boosting CD8+ T cell activity. Consequently, tumors in UFL1 cKO mice responded more favorably to anti-CTLA-4 immunotherapy (134). Another study showed that PD-L1 underwent UFMylation, leading to destabilizing PD-L1 by promoting its ubiquitination. Disrupting PD-L1 UFMylation through the silencing of UFL1 or UFM1 resulted in PD-L1 stabilization in a variety of human and mouse cancer cell lines, which weakened antitumor immunity both *in vitro* and in mouse models. Reduced UFL1 levels were observed across several cancers, and lower UFL1 expression was associated with a diminished response to anti-PD1 therapy in melanoma patients. An inhibitor of UFSP2 enhances UFMylation activity and displayed potential to augment PD-1 blockade therapy (135).

## PROTACs target PD-1/PD-L1

**Figure 2:**
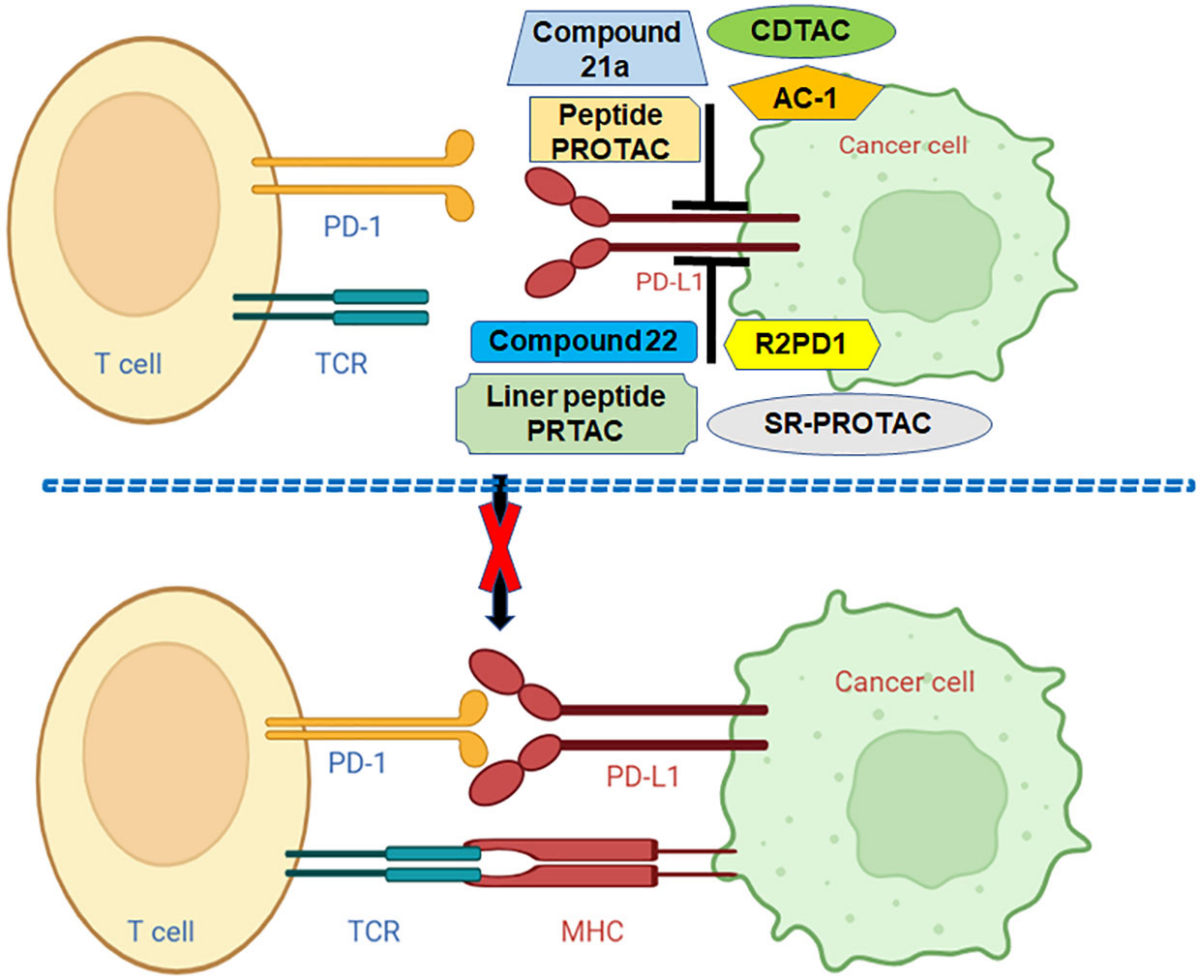
Various PROTACs target PD-L1 in human cancer. Linear peptide PROTAC, SP-PROTAC, R2PD1, Peptide-PROTACs, Compound 21a, CDTACs, AC-1, and Compound 22 target and decrease the PD-L1 and block the PD-1/PD-L1 pathway.

Numerous PROTACs have been developed for targeting PD-L1 and PD-1 (**Table 3**). In the following sections, we will highlight the multiple PROTACs that target PD-1/PD-L1 in human cancers (**Figure 2**).

**Table 3:** PROTACs target PD-1/PD-L1 in cancer.

Compounds	Targets	Functions	Reference
Compound 22	Restores the immunity repressed in CD3 T cells	Restored the immunity repressed in CD3 T cells	(143)
AC-1	Enhances lysosomal degradation of PD-L1 via RNF43 E3 ligase	Improve the immunotherapy	(144)
AbTACs	Efficient degradation of PD-L1 and EGFR	Enhances immunotherapy	(145)
CDTAC	Displays effective PD-L1 degradation	Enhances immune responses via activation of the STINF pathway	(146)
Compound 21a	Induces PD-L1 degradation in the cytoplasm	Increases the invasion ability of CD8+ T cells and retards the tumor growth of MC-38 cells	(147)
Peptide-PROTACs	Targets the degradation of PD-1 and PD-L1	The combination of peptide-PROTACs and cisplatin exhibits a synergistic inhibition on cell proliferation	(148)
R2PD1	Causes PD-L1 degradation in melanoma cells, which is dependent on ZNRF3/RNF43.	Increases cytotoxic ability of T cells, leading to inhibition of tumor cell growth	(149)
SP-PROTAC	Destroys DHHC3 and leads to reduction of PD-L1 in cervical cancer cell lines	Enhances effective on IFN-γ and TNF-α release	(150)
Liner peptide PROTAC	Stimulates DHHC3 degradation, reduces PD-L1 expression in cervical cancer cells	Enhances cisplatin-induced proliferation suppression and IFN-γ and TNF-α release	(151)

### Compound 22 targets PD-L1

Novel resorcinol diphenyl ether-based PROTACs have been designed and demonstrated their inhibitory activity against the PD-1/PD-L1 pathway, and showed their ability to degrade PD-L1 protein. This study used HTRF binding assay to assess the inhibitory activities of PROTACs against PD-1/PD-L1. Most of PROTACs obtained excellent inhibition of PD-1/PD-L1. Compound 22 is one of the best PROTACs with low IC50 value. Moreover, except suppression of PD-1/PD-L1 interaction, compound 22 restored the immunity repressed in CD3 T cells and Hep3B/OS-8/hPD-L1 cells. Compound 22 decreased the expression of PD-L1 protein via lysosome-dependent pathway. Hence, compound 22 could work as a potential agent for the degradation of PD-L1 via PROTAC strategy (143).

### AC-1 targets PD-L1

One group developed antibody-based PROTACs (AbTACs), which are fully recombinant bispecific antibodies. AbTACs degraded cell-surface proteins via recruitment of membrane-bound E3 ligases. AC-1, an AbTAC, enhanced the lysosomal degradation of PD-L1 via recruitment of the RNF43 E3 ligase. Moreover, AC-1 promoted the degradation of PD-L1 after one day treatment in three cancer cell lines, including MDA-MB-231, HCC827, and T24, indicating that AC-1 has wide cellular applicability. Furthermore, due to human parts of AbTAC, AC-1 makes it unlike to illicit an immune response. In summary, AC-1 could be a good AbTAC to target PD-L1 for degradation (144). New AbTACs were engineered for efficient degradation of two membrane proteins, PD-L1 and EGFR (145). In this research, they apply protein engineering techniques to study and enhance the degradation capabilities of PD-L1 and EGFR for therapeutic purposes. They created several antibodies targeting RNF43, discovering that the precise binding sites on RNF43 and the proteins of interest are crucial for degradation effectiveness, which have more binding strength than the AbTAC antibodies. Additionally, this group introduced ZNRF3, another E3 ligase, into our arsenal for degrading PD-L1 and EGFR. Notably, AbTACs targeting RNF43 and ZNRF3 do not trigger WNT signaling pathways. This work suggests that optimizing the development of AbTACs will enhance their utility for targeted protein degradation (145).

### CDTACs target PD-L1

Carbon-dot (CD)-based PROTACs (CDTACs) have been established, which degrade membrane proteins via the UPS pathway. CDTACs were found to interact with PD-L1, and recruit cereblon (CRBN) to induce the ubiquitination and degradation of PD-L1 via proteasomes. Fasting-mimicking diet (FMD) was observed to promote the cellular uptake and proteasome activity. CDTACs treatment led to more than 90% of PD-L1 degradation in CT26 and B16-F10 cancer cells. Furthermore, CDTACs enhanced immune responses via activation of the STINF pathway. Consistently, the combination of CDTACs and FMD treatment suppressed the tumor growth of CT26 and B16-F10 cells. CDTACs displayed effective PD-L1 degradation and activation of immune system (146).

### Compound 21a targets PD-L1

A new PROTAC, compound 21a, has been designed and demonstrated effective functions on the degradation of PD-L1 protein in multiple malignant cells via proteasome pathway, which was in time- and dose-dependent manners. These different cancer cells include MCF-7 breast cancer cell, SW-480 colon cancer cells, PC-3 prostate cancer cells, MB-49 murine bladder tumor cells, and hematological cancer cells (HL-60, Kasumi-1, Skno-1). Compound 21a was designed based on a BMS-37 derivates, which act as small molecule inhibitors against PD-L1. Compound 21a induced PD-L1 degradation in the cytoplasm. *In vivo* experiment data showed that compound 21a increased the invasion ability of CD8+ T cells and retarded the tumor growth of MC-38 cancer cells via reduction of PD-L1 protein levels. Altogether, compound 21a might be an alternative agent for cancer immunotherapy (147).

### Peptide-PROTACs target PD-1/PD-L1

Dai et al. designed Peptide-PROTACs that target PD-1 and PD-L1 in human cancer cells (148). The peptide degraders include a cell-penetrating peptide (CPP) sequence, targeting protein recognition (TPR) peptide sequence, E3 recruitment peptide (ERP) sequence, and peptide linker. Peptides 1 and 2, consisting of VHL binder and CPR sequence, exhibited the highest efficiency for PD-1 and PD-L1 degradation, respectively. Based on TPR sequence, Peptides 1 and 6 targeted PD-1, and Peptide 11 targeted PD-L1. Peptide 1 displayed a higher potent for PD-1 degradation than peptide 6 in C33A cells (148). By AI-direct peptide design, Peptide 2 also targeted PD-L1 palmitoylation. Peptide 12 targeted PD-L1 palmitoylation without ERP sequence. Moreover, Peptide-PROTACs with CPP sequences were found to cross the cell membrane. Furthermore, Peptide-PROTACs displayed a low toxicity to C33A cells. Notably, a combination of Peptide-PROTACs and cisplatin exhibited a synergistic inhibition on cell proliferation via induction of cell apoptosis compared with cisplatin monotherapy (148). Peptide-PROTACs could provide better clinical benefit for cancer patients.

### R2PD1 targets PD-L1

Sun and colleagues discovered ROTACs, which was bispecific WNT- and BMP-signaling-disabled R-spondin (RSPO) chimeras, to influence transmembrane protein degradation via leveraging the specificity of stem cell factors for ZNRF3/RNF43 ligases. R2PD1, a RSPO2 chimera, was uncovered to target the degradation of PD-L1. The R2PD1 protein interacted with PD-L1 and led to its lysosomal degradation. Surprisingly, R2PD1 caused 50% to 90% PD-L1 degradation in melanoma cell lines, which was dependent on ZNRF3/RNF43. Functionally, R2PD1 increased cytotoxic ability of T cells, leading to inhibition of tumor cell growth. Hence, ROTACs represent a new strategy for degrading cell surface proteins (149).

### SP-PROTAC decreases PD-L1

A stapled peptide-based PROTAC (SP-PROTAC) was designed to specifically destroy palmitoyl-transferase (DHHC3), which led to the reduction of PD-L1 in cervical cancer cell lines. In C33A and HeLa cells, SP-PROTAC remarkably reduced PD-L1 protein levels at low concentration. The proteasome inhibitor MG132 attenuated the SP-PROTAC-induced PD-L1 degradation in cancer cells. When T cells and C33A cells were cocultured, SP-PROTAC enhanced IFN-γ and TNF-α release via promotion of PD-L1 degradation. Compared with BMS-8, an ICI, SP-PROTAC displayed more effective on IFN-γ and TNF-α release. Taken together, SP-PROTAC targeted DHHC3 and alleviated PD-L1 protein levels in human cervical cancer (150).

### Liner peptide PROTAC decreases PD-L1

A cyclic peptide-based PROTAC was synthesized to stimulate the degradation of DHHC3 palmitoyltransferase, which resulted in reduce PD-L1 expression in cervical cancer cells (151). This cyclic peptide PROTAC consists of disulfide bonds to maintain their structure via keeping the stability of N- and C-termini of the peptide. MG132 can block cyclic peptide PROTAC-induced degradation of PD-L1. Moreover, cyclic peptide PROTAC enhanced cisplatin-induced proliferation suppression in C33A cells. Furthermore, after C33A and T cells were cocultured, cyclic peptide PROTAC blocked the PD-1/PD-L1 binding via enhancement of PD-L1 degradation, eventually contributing to increased secretion of IFN-γ and TNF-α. This cyclic peptide PROTAC promotes anti-PD-L1 activity in cancer cells (151).

## Conclusion

In conclusion, PD-1 and PD-L1 are regulated by PTMs, including phosphorylation, ubiquitination, deubiquitination, acetylation, palmitoylation, glycosylation and UFMylation. Multiple PROTACs have been developed for targeting PD-L1 and PD-1, including compound 22, AC-1, AbTACs, CDTAC, compound 21a, peptide-PROTACs, R2PD1, SP-PROTAC, liner peptide PROTAC. PROTAC is rapidly evolving and could be a new approach for cancer therapies, including immunotherapy (**Figure 2**). Several issues need to be discussed regarding immunotherapy and PTMs of PD-1/PD-L1. Beyond the abovementioned PTMs, it is unclear whether other types of PTMs regulate PD-1 and PD-L1, which should be determined. Recent studies suggest that miRNAs, lncRNAs and circRNAs are involved in PD-1/PD-L1 expression and tumor immunotherapy (152). LncRNA MALAT1 modulated METTL3-mediated PD-L1 expression and affected immune infiltrates in pancreatic cancer (153). It is unclear whether noncoding RNAs regulate the PD-1/PD-L1 PTMs in human cancer, which needs to be clarified. Besides, PD-1 and PD-L1, whether other immune checkpoint factors are regulated by PTMs and involve in cancer immunotherapy. Although PROTACs have many advantages, such as modularity, targeting on undruggable targets, selectivity, specificity and compatibility, they have several disadvantages, including heavy-molecular weight, unclear pharmacokinetics (67). It is necessary to develop more general strategies for PROTAC-mediated protein degradation. Moreover, it is required to determine whether PROTACs could be used to combine with radiotherapy, chemotherapy and immunotherapy for a wider range of cancers and non-cancer diseases.

## Author contributions

XR: Data curation, Formal analysis, Investigation, Methodology, Project administration, Resources, Software, Writing – original draft. LW: Data curation, Formal analysis, Investigation, Methodology, Project administration, Resources, Software, Writing – original draft. LL: Conceptualization, Formal analysis, Investigation, Project administration, Resources, Supervision, Validation, Visualization, Writing – review & editing. JL: Conceptualization, Project administration, Supervision, Validation, Visualization, Writing – review & editing.

## Glossary

**Table:**

AbTAC	antibody-based PROTAC
CAR-T	Chimeric Antigen Receptor T
c-Cbl	casitas B lymphoma
CDTACs	carbon-dot (CD)-based PROTACs
CRC	colorectal cancer
CRLs	cullin-RING ligases
DUBs	deubiquitinating enzymes
FGFR3	fibroblast growth factor receptor 3
HECT	Homologous to E6-AP carboxyl terminus
HNSCC	head and neck squamous cell carcinoma
ITCH	Itchy E3 Ubiquitin Protein Ligase
JAMM/MPN+	Jab1/MPN domain-containing metalloenzymes
LYTAC	lysosome targeting chimera
Nano-PROTAC	nanoparticle-based PROTAC
NEDD4	Neural Precursor Cell Expressed Developmentally Downregulated 4
NEDD4L	NEDD4-Like
NEDL1	NEDD4-Like E3 Ubiquitin Protein Ligase 1
OUT	ovarian tumor protease
PD-1	programmed death-1
PD-L1	programmed death ligand-1
PROTACs	proteolysis targeting chimeras
PTM	post-translational modification
RBR	RING-between-RING
RING	Really interesting new gene
RLDs	RCC1-like domains
USP	ubiquitin-specific protease
SCF	Skp1-Cullin1-F-box protein
SPOP	speckle-type POZ protein
SP-PROTAC	stapled peptide-based PROTAC
SMURF1	SMAD Specific E3 Ubiquitin Protein Ligase 1
UCH	ubiquitin C-terminal hydrolase
WWP1	WW Domain Containing E3 Ubiquitin Protein Ligase 1.
